# A call to leverage a health equity lens to accelerate human neuroscience research

**DOI:** 10.3389/fnint.2023.1035597

**Published:** 2023-04-17

**Authors:** Vida Rebello, Kristina A. Uban

**Affiliations:** ^1^Department of Health, Society and Behavior, Public Health, Henry and Susan Samueli College of Health Science, University of California, Irvine, Irvine, CA, United States; ^2^Department of Population Health and Disease Prevention, Public Health, Henry and Susan Samueli College of Health Science, University of California, Irvine, Irvine, CA, United States; ^3^Institute for Interdisciplinary Salivary Bioscience Research, University of California, Irvine, Irvine, CA, United States; ^4^Center for the Neurobiology of Learning and Memory, University of California, Irvine, Irvine, CA, United States

**Keywords:** human neuroscience, health equity, social determinants of health (SDoH), health inequities, confounders, counterfactual

## Abstract

Investigation of health inequities tend to be examined, in human neurosciences, as biological factors at the level of the individual. In actuality, health inequities arise, due largely in part, to deep-seated structural factors. Structural inequality refers to the systemic disadvantage of one social group compared to others with whom they coexist. The term encompasses policy, law, governance, and culture and relates to race, ethnicity, gender or gender identity, class, sexual orientation, and other domains. These structural inequalities include but are not limited to social segregation, the intergenerational effects of colonialism and the consequent distribution of power and privilege. Principles to address inequities influenced by structural factors are increasingly prevalent in a subfield of the neurosciences, i.e., cultural neurosciences. Cultural neuroscience articulates the bidirectional relationship between biology and environmental contextual factors surrounding research participants. However, the operationalization of these principles may not have the intended spillover effect on the majority of human neurosciences: this limitation is the overarching focus of the present piece. Here, we provide our perspective that these principles are missing and very much needed in all human neuroscience subdisciplines to accelerate our understanding of the human brain. Furthermore, we provide an outline of two key tenets of a health equity lens necessary for achieving research equity in human neurosciences: the social determinants of health (SDoH) framework and how to deal with confounders using counterfactual thinking. We argue that these tenets should be prioritized across future human neuroscience research more generally, and doing so is a pathway to further gain an understanding of contextual background intertwined with the human brain, thus improving the rigor and inclusivity of human neuroscience research.

## 1. Introduction

Human neuroscience research has experienced remarkable growth, particularly due to technological advancements such as magnetic resonance imaging over the past 50 years. Despite methodological progress, a pressing challenge remains: understanding the impact of historically entrenched policies and principles on neuroscience research, from the inception of scientific inquiries to the dissemination of findings. Cultural neuroscience, a burgeoning field within the human neurosciences, investigates the relationship between human culture and neurobiological processes (Chiao et al., 2010). However, the practices within this branch of neuroscience have not yet led to a spill-over effect on the majority of human neuroscience research, leaving them as exceptional approaches rather than standard procedures in study design and publication. Currently, there is a limited focus on understanding how broader systemic factors influence outcomes related to the human brain. As straightforward as this critique may be, it remains a significant blind spot in today’s current mainstream human neuroscience efforts and perpetuates an on-going barrier in our pursuit to fully understand the human brain. The present perspective presents a call for significantly more attention toward leveraging a health equity lens in human neuroscience research. In doing so, we hope to contribute to the growing literature that echoes this call (Ricard et al., 2022; Webb et al., 2022; La Scala et al., 2023). Once a critical mass sharing these goals among neuroscientists is achieved, we believe a new era of accelerated understanding of the human brain will follow, creating a novel path divergent from the exclusionary practices in scientific history (Rutherford, 2021b).

An overarching aim of human neuroscientific research is to understand how the human brain works. A benefit of this increased understanding is to help people with these novel discoveries. Through increased understanding of the brain, we can better support optimization of neurobiological pathways and their function to promote health and wellbeing while decreasing the prevalence of diseases and disorders. As scientists uniquely situated at the intersection of public health and developmental human neurosciences, we seek to contribute to the accumulating critique of human neuroscience research that clear and problematic blind spots exist and offer our value of leveraging a health equity lens to begin to address these blind spots. In doing so, we acknowledge and operationalize the health equity lens for use in human neuroscience research. We recognize that implementing health equity-focused investigations in the realm of human neuroscience presents considerable challenges, primarily due to the absence of relevant variables within extant neuroscientific data sets. Furthermore, the scarcity of funding opportunities for the creation of new, inclusive data sets and the inherent difficulties in challenging prevailing norms compound these obstacles. Consequently, our current understanding is not exemplary; nonetheless, it represents the most advanced knowledge available at present, and serves as a foundation for initiating individual trajectories aimed at accelerating and broadening the scope of human neuroscience research. The equity lens seeks to embrace the biology-environment interaction of human health and disease. The current challenge in human neurosciences is in expanding to these biology-environment interactions to uncover potential blind spots (Bendesky and Bargmann, 2011). This perspective seeks to give the reader brief examples of societal constructs within the past that contribute to these persistent blind spots in human neuroscience. In doing so, we hope to offer a starting point to human neuroscientists who desire to employ a health equity lens in their research. We write this as both an amendment to the field, as well as encouragement to neuroscientists to consider the intersection of neuroscience and health equity in their own research and be part of the much-needed change in our neuroscience field for the embedment of equitable human neuroscience research.

## 2. Societal constructions of human research

**FIGURE 1:**
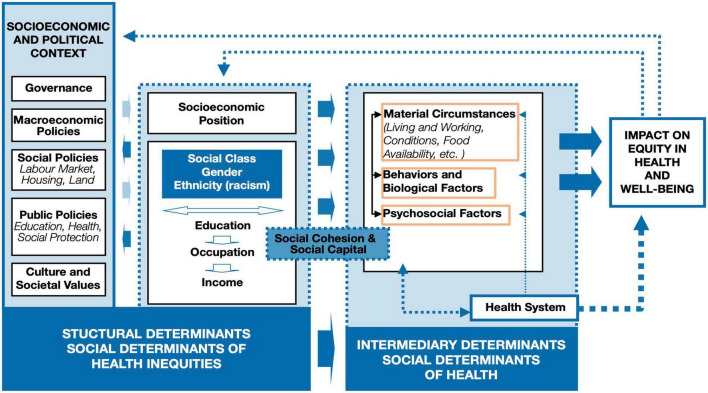
The World Health Organization (WHO) social determinants of health framework structural factors, deeply rooted in imperial policies, influence individual-level factors. Neuroscience often attributes individual brain health outcomes to individual factors (highlighted in orange boxes in the modified figure to emphasize where the majority of factors examined in the human neuroscience research fall; e.g., behaviors and biological factors, psychosocial factors), yet this diagram demonstrates that individual factors are only a small piece of the story. Individual factors are shaped, in part, by the larger socioeconomic and political context; thus, these larger contextualizing structural factors are largely missing from human neuroscientific research at present. Originally published by the World Health Organization (Solar and Irwin, 2010) on page 6 in A conceptual framework for action on the social determinants of health. Permission was granted to reproduce this diagram.

A health equity lens is quick to recognize and elucidate how large proportions of health issues stem not solely from the individual, but also from the structures surrounding the individual. Current structures that drive ongoing inequities were laid by imperialist roots (Roy, 2018). Historical structural factors set the tone for the present-day environment in which human neuroscience research operates: mainly intergenerational wealth- and privilege- dominated (Abiodun, 2019). One such example of historical context leaving lasting impact on today’s structural resources is colonialism, which has resulted in lasting changes in power and resources for entire communities that pesrist today (Czyzewski, 2011; Sherwood, 2014; Araújo et al., 2020). These historical structural factors continue to influence current contextual factors surrounding brain health and may have implications for neurodevelopmental and psychiatric disorders in later life (Gajwani and Minnis, 2022). Thus, attributing an individual’s neurobiological outcome solely to individual-level factors (e.g., genetics, biological vulnerabilities, or personal decisions) misses critical contributions of the more significant systemic factors at play, such as resources, power, intergenerational factors, discrimination and autonomy (Gee and Ford, 2011). Most efforts in neuroscience to go beyond individual-level factors tend only to reach intermediary measures (Figure 1 in orange boxes) and is limited in the examination of the surrounding structural factors’ (i.e., governance, macroeconomic policies, social policies, public policies, race and ethnicity, income) impact on the brain. For instance, neuroscience often attributes individual brain health outcomes to individual-level risk (e.g., material circumstances, behavioral and biological factors, psychosocial factors, genetics), yet, here, we challenge this pitfall by demonstrating that structural factors shape these same individual-level risk factors as well as the individual brain health outcomes. Thus, without substantial consideration of these structural factors, we may have erroneously overemphasized the significance of individual-level, and underemphasized structural factors when understanding the human brain.

Progress in human neuroscience depends on understanding these structural factors, so often shaped by imperialist policies and principles. A paradigm shift toward this goal is the remembrance of past injustices and cultivating a “*Just Memory*.” A *Just Memory* is memory work that recalls both one’s own, as well as, the other’s historical background (Nguyen, 2013). Research has demonstrated the detrimental effects of intergeneration trauma, and so, we are a product of generational osmosis (Bezo and Maggi, 2015; Danieli et al., 2016; O’Neill et al., 2016; Berckmoes et al., 2017; Costa et al., 2018; Williams et al., 2018). The implications of integrating health equity with human neurosciences are significant, as it serves to acknowledge the various contextualizing intergenerational structural factors that may lead to neurobiology that underlie risk and resilience for psychological and mental health outcomes. However, this shift requires a complex roadmap: one that we suggest should first be guided by the epistemic deconstruction of population stereotypes. To contextualize the inheritance of inequities within scientific hegemony and offer two key tenets of health equity that may add to the human neurosciences, we invite readers to be an active part of the expansion of inculcating macrolevel structural factors using two key tenets outlined further to begin the needed shift in human neurosciences.

One reason contemporary neuroscience today continues to perpetuate this limited scope in investigation of how structural factors influence neurobiological processes may be due, in part, to epistemic injustices. Here, we define *scientific epistemic injustice* as a cultural injustice that occurs when the concepts and categories by which research participants understand themselves and their world, are replaced by the concepts and categories by the researcher and the research world. In other words, a pitfall for all researchers is to falsely impose their own worldviews in their scientific methodology, viewing the participating community members through assumptions and therefore, often overly narrow lens. With this pitfall, research can end up biased, with worldviews and values held by the research team being falsely emphasized, while those held by the participating community members are misunderstood or overlooked. The prevailing state perpetuates the risk of drawing erroneous and detrimental conclusions in contemporary human neuroscience literature. This persistence may be attributed to the predominance of privilege and power within the field, which originates from historical systemic factors that continue to influence current practices of wealth and privilege establishment and preservation. The broader academic milieu, where research is primarily conducted, reflects these enduring impacts of historical systemic factors (Stewart and Valian, 2018). Unlike other STEM disciplines, human neuroscience delves into the study of the human condition, which is profoundly shaped by present contextual factors related to historical structural determinants. Indeed, numerous scholars have underscored the significant ethical obligations associated with disseminating findings on biological aspects, such as the human brain, as opposed to non-biological research domains, given that public perceptions of brain discoveries tend to be less amendable (Tolwinski, 2019). Those who have benefited from historical systemic factors that continue to drive privilege today tend to hold administrative control over human neuroscience studies (Kim and Sasaki, 2014; Abiodun, 2019). Human brains develop not merely based on individual molecular or genetic events: cultural and structural factors also influence the brain in parallel. As with all things human, inequality and inequity are important, yet under studied, drivers of human brain outcomes, and their functional outcomes such as behavior, cognition, and mental health.

Brain outcomes result from a constellation of factors: some of which are unknown by the researcher, creating blind spots in our attempt to uncover how the brain develops, functions, and retains resilience to disease and disorder. If neuroscience is to address current blind spots, then our discipline needs to effectively recognize epistemic injustices in our research studies. When all key factors remain uncertain, researchers tend to revert to their own worldviews to help scaffold their understanding of the complex research findings on the human brain. When in a position of not knowing how to model these uncertainties in research, it is human nature to experience the tendency to revert to imposing our own worldview onto the population under study or disregard essential factors in the structure and parametrization of our models that are related to the participant (Saini, 2020). Intergenerational forces shape the structure of human societies, partially or wholly defining the outcomes we study. Thus, it is of great importance to factor in the social and environmental drivers present in our society, which often result from downstream and lasting effects of historical structural factors.

### 2.1. Health equity tenet 1: the social determinants of health (SDoH)

There are communities of people that experience the world differently, and these lived experiences are relevant to scientific progress. After studying the social environment of the African-American communities in 1899, Dr. W. E. B. Du Bois recognized the role of sociohistorical contextual factors that played into the daily life of the community and how they defined health behaviors and diseases (Du Bois and Anderson, 2014). In other words, events that are unwitnessed in the present, still define our research today. As Dr. Du Bois summarized it; “A complete study must not confine itself to the group but must especially notice the environment: the physical environment of the city, sections and houses, the far mightier social environment—the surrounding world of custom, wish, whim and thought which envelopes this group and powerfully influences its development” (Du Bois and Anderson, 2014). So, the question arises: *Are we accounting for sociohistorical contextual factors embedded in a community when conducting our own neuroscientific research?* For this, neuroscientists will benefit by thinking about their research through a Social Determinants of Health (SDoH) lens. Thus, we challenge fellow neuroscientists to think beyond bio-physiological measures on the individual level. SDoH requires considerations beyond the individual level, and incorporates the conditions where people are born, live, learn, work, and play (Braveman and Gottlieb, 2014). Application of the SDoH lens more regularly into the neurosciences is needed because neural correlates extend to and from the SDoH. As illustrated by the World Health Organization (WHO) SDoH framework (Figure 1), SDoH (e.g., social protection and socioeconomic resources) can influence our individual-level research findings (Solar and Irwin, 2010). Therefore, it is a disservice to think of neurobiological pathways without integrating these outcomes to socio-historical events that are still relevant in shaping our neurobiology today. The following hypothetical presents a neuroscientific claim in order to demonstrate limited applicability, threats to internal and external validity of the study and violation of epistemic virtues. We posit that this example represents a recurrent pattern in both historical and current human neuroscientific literature:

*Neuroscientists, aware that exposure to “X” adversely affects adult neurophysiological health, seek to investigate the consequences of prenatal exposure to “X” on fetal brain development. They examine brain volumes in children with prenatal exposure to “X” and recruit participants from a population stereotypically considered “high-risk” and disproportionately impacted by exposure to “X.” In doing so, they believe this approach enhances the statistical sensitivity of their experimental design to detect a real lasting impact of exposure to “X” on childhood brain development if one truly exists. Study findings reveal reduced brain volumes and lower IQ scores in children with prenatal exposure to “X” compared to unexposed children. Thus, the researchers conclude that their study demonstrates that prenatal exposure to “X” causes lasting harm on childhood neurological development. The recommendations made from their study include more targeted interventions to specific populations to better protect pregnancies from the potential harm of exposure to “X,” so that individuals can better protect their pregnancies from “X.”*

This hypothetical scenario highlights the limited applicability of the study’s findings due to inadequate generalizability, as it targets a specific population with a historical reputation for being “high-risk.” However, the origin of this historical reputation (e.g., systemic oppression, discriminatory stereotypes, or a history of scientific publications labeling the community as highly impacted by exposure to “X”) is not considered. Thus, the researchers cannot definitively demonstrate that exposure to “X” is the primary cause of brain and cognitive outcomes, as they fail to conceptualize exposure to “X” within broader contextual factors. The targeted “high-risk” population demonstrates potential flaws in experimental design stemming from unchallenged stereotypes and researchers’ epistemic views, leading to bias in study design and interpretation of findings. In doing so, neuroscientists inadvertently create publishable “evidence” that reinforces pre-existing stereotypes and epistemic views rather than challenging them, as advocated in this perspective piece. The narrow focus on “X” can lead to amplified and false conclusions about the magnitude of harm caused by “X.” Contextual factors may partially or entirely account for prenatal exposure to “X”’ and brain alterations. Further, the widespread use of cognitive correlates to demonstrate the functionality of brain alterations remains problematic when following the *status quo* in using IQ data, given the known cultural insensitivities of IQ scores (Frisby and Henry, 2016; Hood et al., 2022). Despite the glaring problematic nature of this approach, it is often the current *status quo* today among neuroscientific investigation in human neuroscience. The outlined issues become even more harmful when exposure to “X” is viewed as the responsibility of the individual, such as a behavioral choice (e.g., lifestyle causing hypertension during pregnancy, or substance use as a teratogen, or obesity), effectively causing such neuroscientific publications to drive shame, blame and stigma toward entire communities. This masks the parallel narrative of historical and current SDoH factors that also impact pregnancy, health, and brain development. Incorporating SDoH conceptualization in human neuroscience can begin to challenge many of these blind spots and harmful practices within today’s *status quo*. By conceptualizing exposure to “X” as a “symptom” of SDoH factors, it is viewed as a correlate or symptom reflecting SDoH factors, rather than the sole cause of brain outcomes. Here, our example illustrates how adopting SDoH conceptualization can more accurately point to historical and cross-generational roots with on-going impact. Without a SDoH framework, the magnitude of the effect of exposure to “X” is erroneously overemphasized, resulting in false conclusions.

### 2.2. Health equity tenet 2: counterfactuals, contexts, and confounders

“Genetics loads the gun but the environment pulls the trigger” (Stern and Kazaks, 2009). Counterfactual theory involves comparing scenarios related to the occurrence of an outcome under contrasting exposure states (Bours, 2021). It seeks to answer whether the outcome would remain the same or differ if an exposed individual had not been exposed. Counterfactual thinking contrasts contextual factors surrounding the outcome, indicating that the exposure under study may be associated with the outcome in one scenario but not in another. This approach seeks to equalize the background risks associated with research participants, ensuring comparability. To illustrate the application of counterfactual thinking to, for example, colonialism and its downstream effects, requires one to employ a *Just Memory*. This involves understanding the research participants’ sociohistorical background to adequately adjust for confounding variables. Historical contexts can still influence current contexts, making them vital for interpreting results from human neuroscience research. Utilizing a counterfactual framework can help integrate historical and current contextual factors in neuroscience research. For example, within the counterfactual framework, considering colonialism in human neuroscience involves contrasting the lived intergenerational experiences of research participants under colonialism and its oppressive policies against the experiences they would have had without the exposure to coloniality. Quantifying this contrast necessitates understanding confounding variables within the study design.

Confounding refers to outcome differences resulting from variations in the baseline risks of comparison groups (Brooke and Finlayson, 2022). Confounding variables affect the primary relationship under study, leading to spurious associations. Essentially, confounding introduces ambiguity within counterfactual scenarios. Accounting for confounding partially addresses the lived experiences of health inequities among research participants. Moreover, employing an SDoH framework is crucial to satisfactorily account for participants’ lived experiences. By examining counterfactuals and confounders in the context of socially constructed determinants, we can better integrate basic sciences to understand and translate meaningful results for our communities. We emphasize the importance of studying both neurobiological outcomes and the participants’ environment, not in isolation but in conjunction with prevalent social fissures. For example, when investigating the impact of parenting on childhood brain outcomes, not only do parallel and co-occurring confounders likely play a role (e.g., current experienced stressors, racism, resources), but also historical factors that have led to current co-occurring confounders and may serve as counterfactuals (e.g., historical slavery, structural racism like redlining, discrimination-based incarceration, race-based incarceration of a co-parent, geographical food deserts, experienced parenting styles under historical extreme trauma/stressors, among others). Consider the following intuition pump, revolving around counterfactuals and confounders, envision two hypothetical scenarios: Scenario A, in which colonialism played a significant role in shaping the course of history and the development of human neurosciences, and Scenario B, in which colonialism never occurred. In Scenario A, colonial powers exerted control over vast territories, imposing their scientific paradigms, language, and culture on colonized populations. Consequently, the development of human neurosciences was heavily influenced by the dominant scientific paradigms and methodologies of the colonizers. In Scenario B, societies developed independently, with diverse cultures and knowledge systems contributing to the growth of human neurosciences. This scenario would feature a more equitable distribution of scientific contributions and a richer understanding of the human brain and its functions, derived from various cultural perspectives and intellectual traditions. Some aspects influenced by colonialism include but are not limited to:

• Eurocentric perspectives: Colonial powers promoted their own scientific paradigms and methodologies, often disregarding or undermining the knowledge systems and practices of colonized populations. As a result, the development of human neurosciences has largely been shaped by Eurocentric perspectives, which may have limited the inclusion of diverse viewpoints and methodologies.
• Language and communication: The colonizers imposed their languages on colonized territories, leading to the dominance of these languages in scientific research and communication. Consequently, human neuroscience research conducted in non-European languages may have been overlooked or undervalued, resulting in a potential loss of valuable insights and knowledge.
• Access to resources and funding: Colonial powers often controlled the allocation of resources and funding for scientific research, favoring their own scientific agendas and priorities. This has led to the development of human neurosciences being heavily skewed toward the interests and perspectives of the colonizers, while neglecting or marginalizing the research interests of the colonized populations.
• Education and training: Colonizers established educational institutions and training programs in the colonized territories, often modeled after their own systems. These institutions and programs emphasized the scientific paradigms and methodologies of the colonizers, further reinforcing their dominance in the field of human neurosciences.
• Research ethics and practices: The development of human neurosciences under colonial influence may have been accompanied by ethical issues and questionable research practices, including the exploitation of colonized populations as research subjects without proper informed consent or the disregard for cultural sensitivities and values.
• Dissemination of knowledge: The scientific knowledge generated by the colonizers was often disseminated through their own channels, such as scientific journals and conferences, which may have limited the accessibility and visibility of research conducted by non-European scientists or those from colonized territories.

Now, consider the confounders—factors that may influence the relationship between colonialism and the development of human neurosciences. These factors could include economic systems, access to resources, technological advancements, and socio-political dynamics, among others. In both scenarios, these confounders might lead to disparities in the development and dissemination of scientific knowledge.

By comparing Scenario A and Scenario B, we can better understand the impact of colonialism on human neurosciences and the potential benefits of integrating diverse knowledge systems. This thought experiment highlights the importance of considering counterfactuals and confounders when examining the complex relationship between colonialism and scientific development. It encourages reflection on the biases and limitations present in our current understanding of human neurosciences and urges consideration of how we might move toward a more inclusive and representative approach to scientific inquiry. By acknowledging these influences, the scientific community can work toward a more inclusive and representative approach to human neuroscience research, which recognizes the value of diverse knowledge systems and encourages collaboration among researchers from varied research disciplines.

## 3. Conclusion

While human experiences are dynamic, it is grounded in persistent structural factors largely related to imperialist policies and principles, which continue to have a strong hold on human neuroscience, and our pursuit to study it. Historical eugenic policies are rooted in present-day human neuroscience methodology, mislabeled but still trickle down to the principles in how we measure the human brain, accounting for covert misinterpretations [For further reading, we highly recommend the works of Gould (1978), Gee et al. (2019), Ford (2020), and Rutherford (2021a)]. For example, a theory conceptualized by Samuel Morton in the 19th century that anthropometric cranial measurements determine intelligence persists today despite wide opposition (Mitchell, 2018). Human neuroscience has largely overlooked decades-to-centuries of mismeasurement born out of oppression, power, and privilege, and how these have impacted the context in which human neuroscience research is conducted, by whom, with whom and for whom. Moving the needle in human neurosciences will need intentional and collaborative effort to effectively avoid epistemic injustices, apply a SDoH lens, and address counterfactuals and confounding variables as they arise in our own neuroscience research (Carter et al., 2022). Along with these two key tenets of health equity and the growing literature (Carter et al., 2022; Girolamo et al., 2022; Green et al., 2022; Ricard et al., 2022; Webb et al., 2022) that call to expand our understanding of how larger contextualizing structural factors drive persistent brain health inequities, human neuroscience has the potential to move the needle toward authentic justice, equity, diversity, and inclusion, while also accelerating our pursuit to study the human brain.

## Data availability statement

The original contributions presented in this study are included in the article/supplementary material, further inquiries can be directed to the corresponding authors.

## Author contributions

VR and KU contributed to the conception. VR took the lead in writing and editing the manuscript. KU contributed to critical revisions and editing of this manuscript. Both authors contributed to the article and approved the submitted version.

## References

[B1] Abiodun S. J. (2019). “Seeing Color,” a discussion of the implications and applications of race in the field of neuroscience’. *Front. Hum. Neurosci.* 13:280. 10.3389/FNHUM.2019.00280/BIBTEX PMC6700357 31456674

[B2] Araújo C. A. Ray J. G. Figueiroa J. N. Alves J. G. (2020). “BRAzil magnesium (BRAMAG) trial: a double-masked randomized clinical trial of oral magnesium supplementation in pregnancy.” *BMC Pregn. Child.* 20:1–7. 10.1186/s12884-020-02935-7 32316938 PMC7175576

[B3] Bendesky A. Bargmann C. I. (2011). Genetic contributions to behavioural diversity at the gene-environment interface. *Nat. Rev. Genet.* 12, 809–820. 10.1038/NRG3065 22064512

[B4] Berckmoes L. H. Eichelsheim V. Rutayisire T. Richters A. Hola B. (2017). “How legacies of genocide are transmitted in the family environment: a qualitative study of two generations in rwanda”. *Societies* 7:24. 10.3390/SOC7030024

[B5] Bezo B. Maggi S. (2015). “Living in “survival mode:” intergenerational transmission of trauma from the holodomor genocide of 1932–1933 in Ukraine”. *Soc. Sci. Med.* 134 87–94. 10.1016/J.SOCSCIMED.2015.04.009 25931287

[B6] Bours M. J. L. (2021). ‘Tutorial: a nontechnical explanation of the counterfactual definition of effect modification and interaction’. *J. Clin. Epidemiol.* 134 113–124. 10.1016/J.JCLINEPI.2021.01.022 33548464

[B7] Braveman P. Gottlieb L. (2014). “The social determinants of health: it’s time to consider the causes of the causes”. *Public Health Rep.* 129:19. 10.1177/00333549141291S206 24385661 PMC3863696

[B8] Brooke B. S. Finlayson S. R. G. (2022). “Critical assessment of surgical outcomes and health services research – ClinicalKey,” in *Sabiston book of surgery*, 21st Edn, ed. Townsend C. (Amsterdam: Elsevier), 159–169. Available online at: https://www.clinicalkey.com/#/content/book/3-s2.0-B9780323640626000086 (accessed August 29, 2022).

[B9] Carter S. E. Mekawi Y. Harnett N. G. (2022). “It’s about racism, not race: a call to purge oppressive practices from neuropsychiatry and scientific discovery”. *Neuropsychopharmacology* 2022 1–2. 10.1038/s41386-022-01367-5 35773434 PMC9630507

[B10] Chiao J. Y. Hariri A. R. Harada T. Mano Y. Sadato N. Parrish T. B. (2010). “Theory and methods in cultural neuroscience”. *Soc. Cogn. Affect. Neurosci.* 5 356–361. 10.1093/scan/nsq063 20592044 PMC2894689

[B11] Costa D. L. Yetter N. Desomer H. (2018). ‘Intergenerational transmission of paternal trauma among US civil war ex-POWs’. *Proc. Natl. Acad. Sci. U.S.A.* 115 11215–11220. 10.1073/PNAS.1803630115/SUPPL_FILE/PNAS.1803630115.SAPP.PDF 30322945 PMC6217388

[B12] Czyzewski K. (2011). Colonialism as a broader social determinant of health. *Int. Indig. Policy J.* 2:215. 10.18584/IIPJ.2011.2.1.5

[B13] Danieli Y. Norris F. H. Engdahl B. (2016). “Multigenerational legacies of trauma: modeling the what and how of transmission”. *Am. J. Orthopsy.* 86 639–651. 10.1037/ORT0000145 26765546

[B14] Du Bois W. E. B. Anderson E. (2014). A social study *“The philadelphia negro”*. (University of Pennsylvania Press). 10.9783/9780812201802

[B15] Ford C. L. (2020). “Commentary: addressing inequities in the era of COVID-19: the pandemic and the urgent need for critical race theory”. *Family Commun. Health* 43 184–186. 10.1097/FCH.0000000000000266 32427666

[B16] Frisby C. L. Henry B. (2016). Science, politics, and best practice: 35 years after larry p. *Contemp. School Psychol.* 20 46–62. 10.1007/S40688-015-0069-3

[B17] Gajwani R. Minnis H. (2022). “Double jeopardy: implications of neurodevelopmental conditions and adverse childhood experiences for child health”. *Eur. Child Adoles. Psychiatry* 1 1–4. 10.1007/S00787-022-02081-9/FIGURES/1 PMC9908716 36156745

[B18] Gee G. C. Ford C. L. (2011). “Structural racism and health inequities: old issues. New directions”. *Soc. Sci. Res. Race* 8 115–132. 10.1017/S1742058X11000130 25632292 PMC4306458

[B19] Gee G. C. Hing A. Mohammed S. Tabor D. Williams D. (2019). Racism and the life course: taking time seriously. *Am. J. Public Health* 109 S43–S47. 10.2105/AJPH.2018.304766 30699016 PMC6356137

[B20] Girolamo T. Parker T. C. Eigsti I. M. (2022). “Incorporating dis/ability studies and critical race theory to combat systematic exclusion of black, indigenous, and people of color in clinical neuroscience”. *Front. Neurosci.* 16:1533. 10.3389/FNINS.2022.988092/BIBTEX PMC9495932 36161181

[B21] Gould S. J. (1978). “Morton’s ranking of races by cranial capacity. Unconscious manipulation of data may be a scientific norm”. *Science* 200 503–509. 10.1126/SCIENCE.347573 347573

[B22] Green K. H. Groep I. H. Brinke L. W. Cruijsen R. Rossenberg F. Marroun H. E. (2022). “A perspective on enhancing representative samples in developmental human neuroscience: connecting science to society”. *Front. Integrat. Neurosci.* 16:111. 10.3389/FNINT.2022.981657/BIBTEX PMC9480848 36118120

[B23] Hood E. Boone K. Miora D. Cottingham M. Victor T. Zeigler E. (2022). Are there differences in performance validity test scores between African American and white American neuropsychology clinic patients? *J. Clin. Exp. Neuropsychol.* 44 31–41. 10.1080/13803395.2022.2069230 35670549

[B24] Kim H. S. Sasaki J. Y. (2014). “Cultural neuroscience: biology of the mind in cultural contexts”. *Annu Rev. Psychol.* 65 487–514. 10.1146/ANNUREV-PSYCH-010213-115040 24050186

[B25] La Scala S. Mullins J. L. Firat R. B. (2023). Equity, diversity, and inclusion in developmental neuroscience: Practical lessons from community-based participatory research. *Front. Integr. Neurosci.* 16:141. 10.3389/FNINT.2022.1007249 37007188 PMC10060815

[B26] Mitchell P. W. (2018). The fault in his seeds: lost notes to the case of bias in samuel george morton’s cranial race science. *PLoS Biol.* 16:7008. 10.1371/journal.pbio.2007008 30286069 PMC6171794

[B27] O’Neill L. Fraser T. Kitchenham A. McDonald V. (2016). “Hidden burdens: a review of intergenerational, historical and complex trauma, implications for indigenous families”. *J. Child Adoles. Trauma* 11 173–186. 10.1007/S40653-016-0117-9 32318148 PMC7163829

[B28] Nguyen V. T. (2013). “just memory: war and the ethics of remembrance”. *Am. Literary History* 25 144–163. 10.1093/ALH/AJS069

[B29] Ricard J. A. Parker T. C. Dhamala E. Kwasa J. Allsop A. Holmes A. J. (2022). “Confronting racially exclusionary practices in the acquisition and analyses of neuroimaging data”. *Nat. Neurosci.* 26 4–11. 10.1038/s41593-022-01218-y 36564545 PMC12884511

[B30] Roy R. D. (2018). *Science still bears the fingerprints of colonialism.* Available online at: https://www.smithsonianmag.com/science-nature/science-bears-fingerprints-colonialism-180968709/ (accessed August 18, 2022).

[B31] Rutherford A. (2021a). “A cautionary history of eugenics”. *Science* 373:1419. 10.1126/SCIENCE.ABM4415 34554795

[B32] Rutherford A. (2021b). “Race, eugenics, and the canceling of great scientists”. *Am. J. Phys. Anthropol.* 175 448–452. 10.1002/AJPA.24192 33332589

[B33] Saini A. (2020). “Want to do better science? Admit you’re not objective”. *Nature* 579:175. 10.1038/D41586-020-00669-2 32152605

[B34] Sherwood J. (2014). “Colonisation – it’s bad for your health: the context of Aboriginal health”. *Contemp. Nurse* 46 28–40. 10.5172/CONU.2013.46.1.28 24716759

[B35] Solar O. Irwin A. (2010). A conceptual framework for action on the social determinants of health. *Soc. Determ. Health Dis.* 2010:852.

[B36] Stern J. Kazaks A. (2009). *Obesity: A reference handbook*. Santa Barbara, CA: ABC-CLIO, LLC, 322.

[B37] Stewart A. J. Valian V. (2018). An inclusive academy: achieving diversity and excellence. *Inclus. Acad.* 10.7551/MITPRESS/9766.001.0001.

[B38] Tolwinski K. (2019). “Fraught claims at the intersection of biology and sociality: managing controversy in the neuroscience of poverty and adversity”. *Soc. Stud. Sci.* 49 141–161. 10.1177/0306312719839149 30917764

[B39] Webb E. K. Cardenas-Iniguez C. Douglas R. (2022). “Radically reframing studies on neurobiology and socioeconomic circumstances: a call for social justice-oriented neuroscience”. *Front. Integrat. Neurosci.* 16:104. 10.3389/FNINT.2022.958545/BIBTEX PMC9479322 36118113

[B40] Williams M. T. Printz D. M. B. DeLapp R. C. T. (2018). “Assessing racial trauma with the trauma symptoms of discrimination scale”. *Psychol. Viol.* 8 735–747. 10.1037/VIO0000212

